# Annotations

**Published:** 1905-04-29

**Authors:** 


### ANNOTATIONS.

### Vaccination Dues and Difficulties.

The report of the Committee on Vaccination Expenses will be read with interest by all who are concerned with this important department of public health. The present system has done much to make vaccination more reliable and satisfactory, and thereby to remove much misapprehension. But the increased efficiency has meant increased cost, and the committee sat to inquire if these costs were in all cases as low as might be expected. On the whole they answer the question in the affirmative, though they make some suggestions which are eminently reasonable. The present system takes no account of the distances which a public vaccinator may have to travel. To meet this difficulty the committee suggest a sliding scale of fees, the minimum one of half-a-crown being paid when the vaccinator does his work in his own office, and rising with the distance he has to travel up to 5s. for distances over two miles. This, however, is not to be a maximum, and when the journey exceeds three miles, special terms may be arranged. This plan would help to hold the balance more evenly between town vaccinators and country ones than has hitherto been the case with the fixed fee system. The committee also suggests that where there is much vaccinating to do, it might be better for Boards of Guardians to appoint vaccinators at a fixed salary who would give their whole time to the work. Naturally such an appointment would be possible only in the more crowded districts of towns, and could not in any case be immediately introduced. In many cases the district medical officers act also as public vaccinators, and regard the vaccination fees as a necessary addition to the small remuneration they receive from the work of parish doctor. The committee point out that, while the vaccination officer receives a fee for every primary vaccination effected, he has much clerical work to do in cases of "postponement," "insusceptibility," and "exemption," for which he receives no remuneration, and their recommendation that the postages of vaccination officers should be paid, and that they should receive a fee of 3d. for the registration of certificates of insusceptibility and postponement is therefore very reasonable.

### Departmental Anodyne.

The sedative influence exercised on popular agitation by the appointment of a Royal Commission or Departmental Committee is a commonplace of political experience. It has the apparent virtue of "doing something," and often also succeeds in shelving awkward questions until popular attention is occupied in other directions. One illustration of these results can readily be found in the recent history of the discussion of the physical condition of the British peoples. At the outset great popular interest was aroused and maintained by the usual series of sensational statements in the public press. There was a real and widespread concern that the country was rapidly going to the dogs, and the authorities were urged and pressed to take action. As a result an inquiry by a Departmental Committee was instituted. The report issued by this Committee certainly showed that many of the statements which had been made on the subject of the physical deterioration of the nation were grossly overcoloured and exaggerated, and that for others there was no adequate body of supporting evidence. But this was not by any means the whole of the report, though it is the part on which emphasis has been laid by many writers for the Press. Hence, whilst the Committee, as a matter of fact, found that it is impossible to say whether the physical condition of the inhabitants of the British Isles is or is not undergoing deterioration, as no records of measurements exist, they have appeared to the public mainly as smooth prophets and as successful and official opponents of the alarmists. Of all their recommendations in reference to the supervision and statistical registration of school children, and of the influence of residence in slum property, and of various occupations on physical development, not one appears to have excited even a measure of public attention. Calm has returned to the public mind largely because those who first alarmed the country by sensational statements now assure us that we are not so black as we were painted.

### Public Health and the Poor-law.

In a lecture on this subject recently delivered to the Royal Philosophical Society of Glasgow, Dr. A. R. Chalmers discussed the relationships of sanitation in regard to social problems generally. He pointed out that the evils with which sanitary legislation is designed to cope have long been recognised, but the motive for the institution of that legislation is provided by an appreciation of the fact that disease, in addition to its power of prejudicing the individual, is a factor in national welfare. As a result of this there has been called into existence, in connection with certain of the problems of disease, forces which are social and political rather than purely medical. The causation of disease is in many instances found to be dependent on conditions capable of alteration at will. These conditions come within the contemplation of various public authorities and administrative methods, and though differentiation of function has its advantages in the body politic it may be a fair question whether at the present day there is not danger of losing sight of the essential unity of the question to which one and all are alike related. It seems desirable, if possible, to devise some plan of supervision under which co-ordination of the many functions of local administration can be secured. For example, the functions of the poor-law and of sanitary administration exist wholly without relation in their executive branches. Yet one of the most commanding causes of poverty is disease, and the prevention of the one has, therefore, the closest possible relationship to the prevention of the other. Dr. Chalmers suggests the selection of an area for the application of all the agencies dealing with sanitary and related problems. Study on such a plan would be a scheme of co-operative clinical observation of disease in the social organism, and the lessons so acquired could hardly fail to be of much value.

